# New plasma preparation approach to enrich metabolome coverage in untargeted metabolomics: plasma protein bound hydrophobic metabolite release with proteinase K

**DOI:** 10.1038/s41598-018-27983-0

**Published:** 2018-06-22

**Authors:** Renata Wawrzyniak, Anna Kosnowska, Szymon Macioszek, Rafał Bartoszewski, Michał Jan Markuszewski

**Affiliations:** 1 Department of Biopharmaceutics and Pharmacodynamics, Medical University of Gdańsk, Gdańsk, Poland; 2 Department of Biology and Pharmaceutical Botany, Medical University of Gdańsk, Gdańsk, Poland

## Abstract

Plasma untargeted metabolomics is a common method for evaluation of the mechanisms underlying human pathologies and identification of novel biomarkers. The plasma proteins provide the environment for transport of hydrophobic metabolites. The current sample preparation protocol relies on the immediate precipitation of proteins and thus leads to co-precipitation of a significant fraction of hydrophobic metabolites. Here we present a new simple procedure that overcomes the co-precipitation problem and improves metabolome coverage. Introducing an additional step preceding the protein precipitation, namely limited digestion with proteinase K, allows release of associated metabolites through the relaxation of the native proteins tertiary structure. The modified protocol allows clear detection of hydrophobic metabolites including fatty acids and phospholipids. Considering the potential involvement of the hydrophobic metabolites in human cardiovascular and cancer diseases, the method may constitute a novel approach in plasma untargeted metabolomics.

## Introduction

The untargeted metabolomic approach, constitutes one of the most frequently applied method in metabolomics studies. It aims to measure the comprehensive metabolomic profiles of various biological samples. The main goal of the application of the untargeted approach in biomedical studies is to discover novel biological markers as well as to gain new insights into mechanisms underlying the pathophysiology of human diseases^1^. However, due to the diversity of physicochemical properties and concentration range of metabolites present in biological samples, the use of complementary analytical techniques is required to provide proper metabolome coverage. Among currently available analytical platforms, liquid chromatography coupled with mass spectrometry (LC-MS) allows determination of the highest number of metabolites^2,3^. LC-MS is a suitable technique for determination of non-volatile, thermally unstable, high- or low-molecular-weight compounds representing a wide range of polarity. Additionally, the widespread availability and continuous development of instrumentation have resulted in extensive applications of the LC-MS technique in untargeted metabolomics^4^.

Notably, due to its near non-invasive sampling methodology and reflection of global metabolic response to different stimuli, such as disease progression, applied pharmacotherapy or diet, genetic modification and environmental factors, metabolomics research is currently dominated by the analysis of blood plasma and urine samples.

Given the fact that the successful application of untargeted metabolomics relies on efficient determination of the widest possible range of metabolites in biological matrices, sample preparation constitutes the crucial step in the methodological workflow since it may affect metabolite content, data quality and interpretation of any obtained results. The choice of sample preparation method mainly depends on the sample type and volume, physicochemical properties of the measured analytes as well as the analytical platform used for sample analysis^5,6^. The sample preparation procedure for global metabolic profiling should be unselective, simple, fast, reproducible and it should include a metabolism-quenching step to reflect adequate metabolome composition at the time of sample collection as well as to minimize metabolite losses^6^. Without doubt, a properly chosen and optimized sample preparation procedure is a key factor in the reliable evaluation of a comprehensive metabolic profile and biological interpretation of the data.

In the case of LC-MS-based plasma untargeted metabolomics, the commonly used sample preparation approaches include protein precipitation (PP)^7,8^ with various organic solvents, liquid-liquid extraction (LLE)^7^, solid-phase extraction (SPE)^7,8^ or ultrafiltration^7^. Nevertheless, protein fraction removal using organic solvents or a mixture thereof, followed by centrifugation and supernatant filtration, constitutes the most frequently used approach in plasma untargeted metabolomics with the use of the LC-MS technique. This procedure is fast, simple, reproducible and provides good plasma metabolome coverage. Importantly, this common approach accounts for the fact that plasma constitutes a hydrophilic environment and thus limits the solubility of hydrophobic metabolites such as lipids, fatty acids, steroids and thyroid hormones. The efficient transport and distribution of these hydrophobic compounds in plasma *in vivo* is achieved through their interaction with proteins that bind them as substrates, products, cofactors and ligands via relatively strong protein-hydrophobic interactions^9^. Indeed, the most abundant human plasma protein, serum albumin, besides maintaining osmotic pressure, acts as a carrier of hydrophobic hormones, fatty acids and lipids as well as binds toxic substances and drugs^10^. Furthermore, lipoproteins govern the specific transport of cholesterol and phospholipids. Finally, plasma immunoglobulins bind antigens that are often insoluble in a hydrophilic environment to mediate their removal^10^.

Hence, the immediate plasma protein precipitation during untargeted metabolomics procedures leads to co-precipitation of a significant fraction of plasma metabolites that results in their respective intensities being below the limit of detection (LOD). The ablation of these metabolites during the PP step can be a limiting factor for disease related metabolomic analyses. This highlights the necessity for optimization of sample preparation methods to include this plasma protein-associated metabolite fraction. Thus, the main goal of this study was to develop and apply a new simple plasma preparation approach which may overcome the co-precipitation problem and improve metabolome coverage. An additional step, aiming to misfold the native proteins and their complexes to release strongly associated metabolites, such as plasma limited digestion with proteinase K (PK), prior to the global protein fraction precipitation was included in the proposed procedure. PK is a well-known proteolytic enzyme that belongs to the serine protease class produced by *Tritirachium album*^11^. This enzyme possesses a broad cleavage specificity and breaks the peptide bond adjacent to the carboxylic group of aliphatic and aromatic amino acids which constitutes a useful feature for protein digestion in biological samples^12^. PK is active over a wide pH range, namely 4.3–12.0. The maximum activity of the enzyme is observed at the temperature of 37 °C and requires the presence of calcium ions^12^. Furthermore, the stability of PK in the presence of anionic surfactants including sodium dodecyl sulfate (SDS) and its ability to digest native proteins mean that this enzyme is commonly used in a variety of applications such as preparation of chromosomal DNA for pulsed-filed gel electrophoresis, protein fingerprinting and removal of nucleases from DNA and RNA preparations^12^.

In the present study, a commonly applied plasma preparation procedure was modified with an additional step including incubation with PK. Additionally, various organic solvents for PP in LC-MS-based plasma non-targeted metabolomics were used and evaluated. The developed and optimized approach was tested for plasma samples derived from urogenital tract cancer patients.

The proposed new and optimized procedure increased the number as well as signal intensity of putatively identified endogenous metabolites. Furthermore, the proteinase K procedure allows detection of individual metabolites in plasma samples, significantly enriching plasma metabolome coverage. To the best of the authors’ knowledge, this is the first study to apply this new plasma preparation procedure for LC-MS-based untargeted metabolomics. The proposed methodology may provide an enrichment of plasma metabolome coverage, mainly in terms of metabolite groups such as phospholipids, sphingolipids, fatty and bile acids, acylcarnitines and amino acids.

Finally, enriching the procedure with the PK digestion step allows clear detection of hydrophobic metabolites including, among others, hydrophobic amino acids, fatty acids and phospholipids. Given the potential involvement of hydrophobic metabolites in human cardiovascular and cancer diseases, the method proposed here may constitute a novel approach in related metabolomics analysis.

## Materials and Methods

### Collection of plasma samples

Blood samples derived from patients treated in the Department of Urology and Department of Family Medicine of Medical University of Gdańsk. Blood samples were collected in accordance with the guidelines of the independent ethics committee at the Medical University of Gdansk (NKBBN/61/2017). The experimental protocols were also approved by the independent ethics committee at the Medical University of Gdansk. The informed consent was obtained from all participants of the study. Blood samples were collected into heparin tubes under fasting conditions and, after centrifugation (13 000 × g, 15 min, 4 °C), the obtained plasma samples were frozen at −80 °C before metabolomic analyses. To evaluate application of the newly proposed sample preparation procedure, plasma samples (n = 10) collected from urogenital tract cancer patients treated in the Department of Urology of Medical University of Gdańsk were used.

### Chemicals and reagents

Acetonitrile, ethanol and methanol were purchased from J.T. Baker (Arnhem, Netherlands). The deionized water was obtained with Milli-RO and Milli-QPlus Millipore instrumentation (Zug, Switzerland). 1-(4-fluorobenzyl)-5-oxoproline and reference standards (e.g. oleic acid) were purchased from Sigma Aldrich (St. Louis, MO, USA). Proteinase K was provided by A&A Biotechnology (Gdynia, Poland). Calcium chloride was purchased from Fluka^TM^, (Buchs, Switzerland).

### Plasma preparation procedures

**Figure 1:**
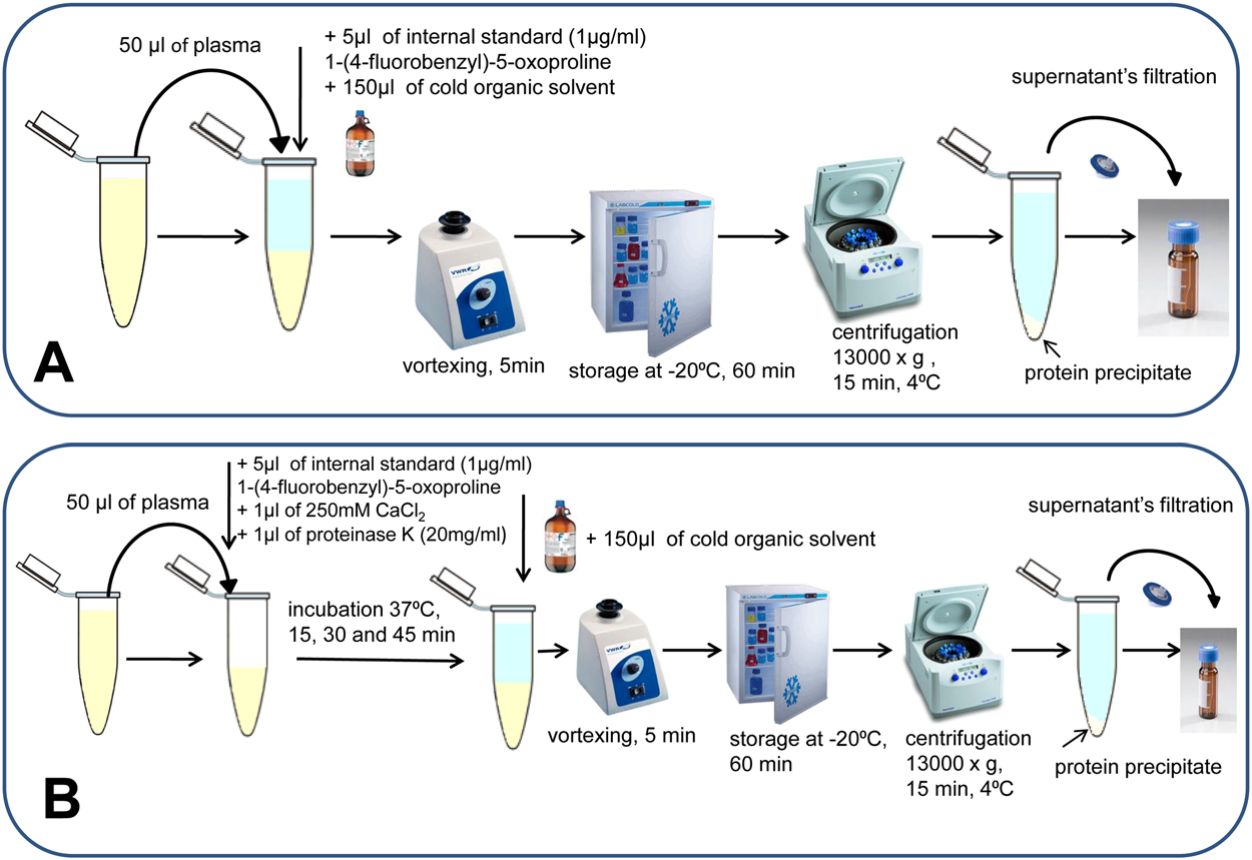
Detailed methodology of different plasma preparation procedures evaluated and compared in the study. **A**: Plasma preparation procedure using various organic solvents for protein precipitation and metabolite extraction. **B**: New plasma preparation approach employing additional incubation with proteinase K before protein precipitation and metabolite extraction with the use of various organic solvents.

Plasma samples were prepared with the use of a commonly applied procedure including protein precipitation, centrifugation and supernatant filtration. Three organic solvents, i.e. acetonitrile, methanol and methanol:ethanol (J.T. Baker, Netherlands) (1:1, v/v), were used in a 3:1 (v/v) ratio with plasma and then compared. The second proposed approach includes addition of proteinase K (A&A Biotechnology, Poland; 1 µl, 20 mg/ml, 6 U) into 50 µl (approx. 2–2.5 mg of total protein) of plasma before the PP step using the above-mentioned organic solvents. Hence, the PK activity was not the limiting factor. To ensure optimal activity of PK, 1 µl of 250 mM CaCl_2_ (Fluka^TM^, Switzerland) was added to 50 µl of plasma sample to obtain a 5 mM final concentration. Different plasma incubation times (15, 30 and 45 minutes) with PK at 37 °C were tested and compared. The detailed methodology for both procedures is graphically presented in Fig. 1. For sample preparation development, six replicates of pooled plasma were used for each tested procedure. Next, the procedure optimized with the use of PK was applied for plasma samples derived from urogenital tract cancer patients and this was then compared with an approach including only protein precipitation.

### Analytical measurements

Plasma metabolic fingerprints were measured with an HPLC system (Agilent Technologies 1200 series, Waldbronn, Germany) coupled with a Time-of-Flight (TOF) mass analyzer (Agilent Technologies 6224 series, Waldbronn, Germany) equipped with a dual electrospray ionization source. The detailed chromatographic and mass spectrometer parameters are described in the Supplementary information section. The prepared plasma samples were analyzed in a randomized order. Two separate sequences were conducted: first in positive and second in negative ionization mode.

### Data processing and metabolite identification

The raw data sets were deconvoluted using MassHunter Qualitative Analysis B.06.00 software (Agilent Technologies, Waldbronn, Germany). The parameters of data extraction were similar to those previously published^13^. To correct the common shift of retention time and measured monoisotopic mass during LC-MS analyses, peak alignment is necessary to mark detected analytical signals as the same features in all measured plasma samples. The alignment step was performed using Mass Profiler Professional B.02.01. (Agilent Technologies, Waldbronn, Germany). After alignment, the obtained data matrices were filtered and normalized using the intensity of the internal standard (IS), namely 1-(4-fluorobenzyl)-5-oxoproline. Only variables present in all plasma samples (n = 6, for each sample preparation procedure evaluated) and with a coefficient of variation (CV) lower than 20% were used for further identification. The filtered analytical signals were putatively identified based on monoisotopic mass, isotopic distribution, formula and hits found in available databases, such as METLIN (www.metlin.scripps.edu), KEGG (www.genome.jp/kegg), LIPIDMAPS (www.lipidmaps.org/), HMDB (www.hmdb.ca) and CEU MassMediator (http://ceumass.eps.uspceu.es/mediator).

## Results

### Comparison of various organic solvents used for plasma protein precipitation and metabolite extraction

Various organic solvents were used and compared, such as acetonitrile, methanol and methanol:ethanol (1:1, v/v) in a 3:1 (v/v) ratio with plasma. The determined plasma metabolomic profiles were evaluated for both the number of extracted features and reproducibility of signal intensities derived from these features. The obtained results are collected and presented in Table S1 in the Supplementary information section. Based on the above-mentioned criteria, the methanol:ethanol mixture (1:1, v/v) provided the most efficient and reproducible metabolite extraction from plasma samples.

### Development and optimization of a new plasma preparation procedure with the use of proteinase K

**Figure 2:**
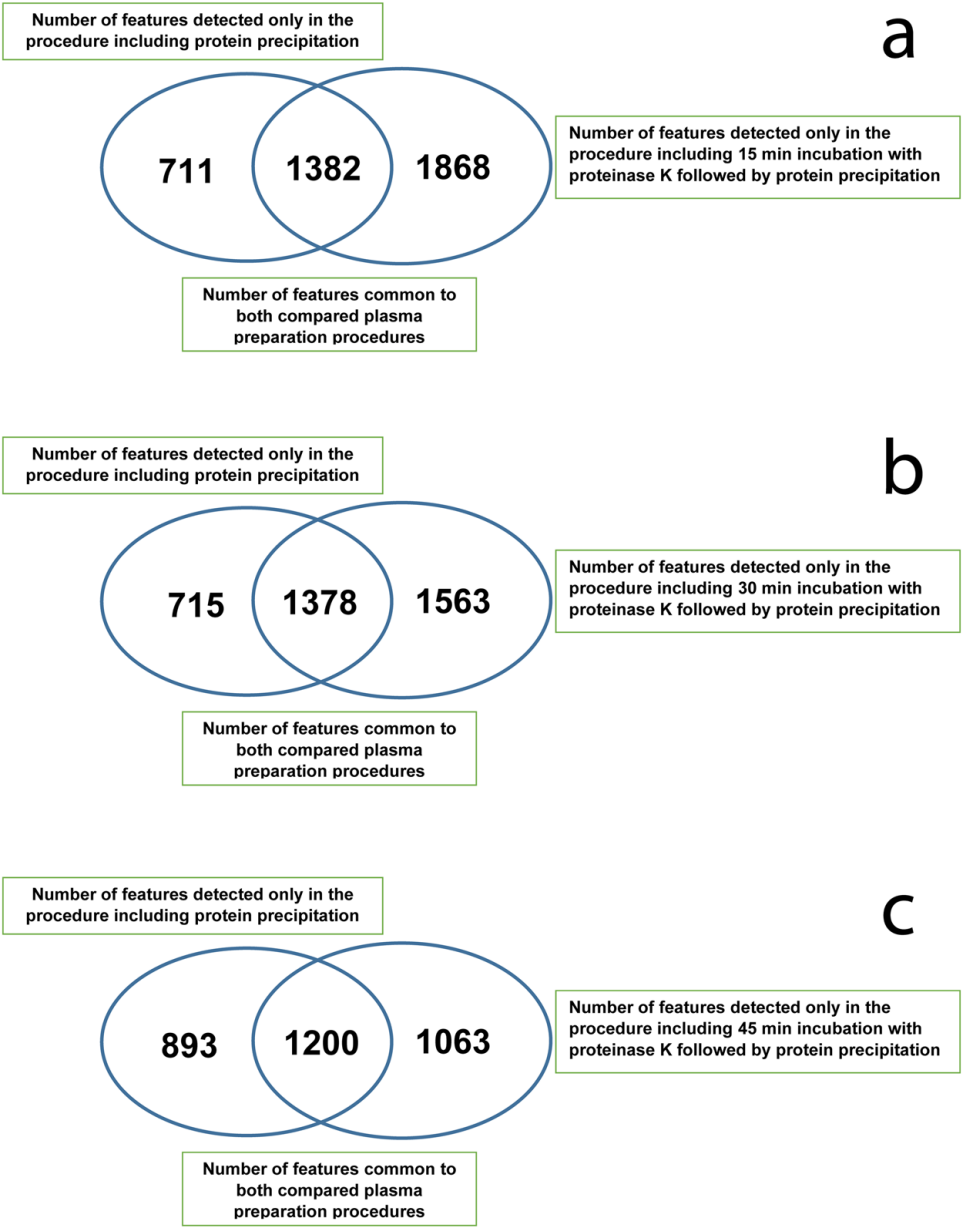
Venn diagrams showing a comparison of the total number of features detected in plasma samples prepared using both tested procedures. (**a**) 15 min incubation for the proteinase K approach, (**b**) 30 min incubation for the proteinase K approach, (**c**) 45 min incubation for the proteinase K approach) Plasma metabolic fingerprints were determined with the LC-ESI-TOF-MS technique in both positive and negative ionization modes.

The commonly applied PP and metabolite extraction approaches using organic solvents were compared with a procedure including additional plasma incubation with PK. Although the new approach with PK was tested and evaluated for all the above-mentioned organic solvents, the results obtained only for the mixture of methanol:ethanol (1:1, v/v) are described and presented here. The results obtained for the comparison of both procedures and different incubation times with proteinase K are presented in Fig. 2. A comparison of results observed for the optimization of the procedure with the use of PK in terms of incubation time is presented in Table 1. The obtained data were evaluated based on the total number measured features, the CV of their intensities, the number of putatively identified endogenous metabolites and the number of peptide fragments.

**Table 1:** Comparison of results obtained for the optimization of the procedure with the use of proteinase K in different incubation times with plasma samples.

	ME15	ME30	ME45
Total number of features present in all plasma samples (n = 6)	3250	2941	2263
Number of features with CV of their intensities < 20%	1456	1047	484
Number of putatively identified endogenous metabolites	582	471	175
Number of peptide fragments	310	179	121

Plasma metabolic fingerprints were measured with the LC-ESI-TOF-MS technique in both positive and negative ionization modes. After plasma incubation with proteinase K, protein precipitation with methanol:ethanol (1:1, v/v) was applied.

ME15: 15 min incubation with proteinase K followed by protein precipitation with methanol:ethanol (1:1, v/v), ME30: 30 min incubation with proteinase K followed by protein precipitation with methanol:ethanol (1:1, v/v), ME45: 45 min incubation with proteinase K followed by protein precipitation with methanol:ethanol (1:1, v/v).

Importantly, the plasma proteins interact with the metabolites mainly through these proteins tertiary structure hydrophobic interaction (not by covalent bounds) and the mild digestion (that leads to the protein misfolding) is required in order to relax the protein structure and release the metabolites. Too intense or complete digestion of plasma proteins will result in their fragmentation into short peptides, that are very difficult to precipitate, and may reaggregate randomly with metabolites. Furthermore, extensive protein digestion affects matrix effect by changing plasma composition, mainly in terms of short peptide fragments. This may result in ion suppression phenomenon. Ion suppression negatively affects several analytical figures of merit, such as ionization efficiency and detection capability. Therefore, the number of detected features may be reduced with increased plasma incubation time with proteinase K (Table 1).

Taken together, too extensive digestion of plasma proteins is the method limiting factor and establishing optimal limited digestion conditions is crucial for the method sensitivity.

**Figure 3:**
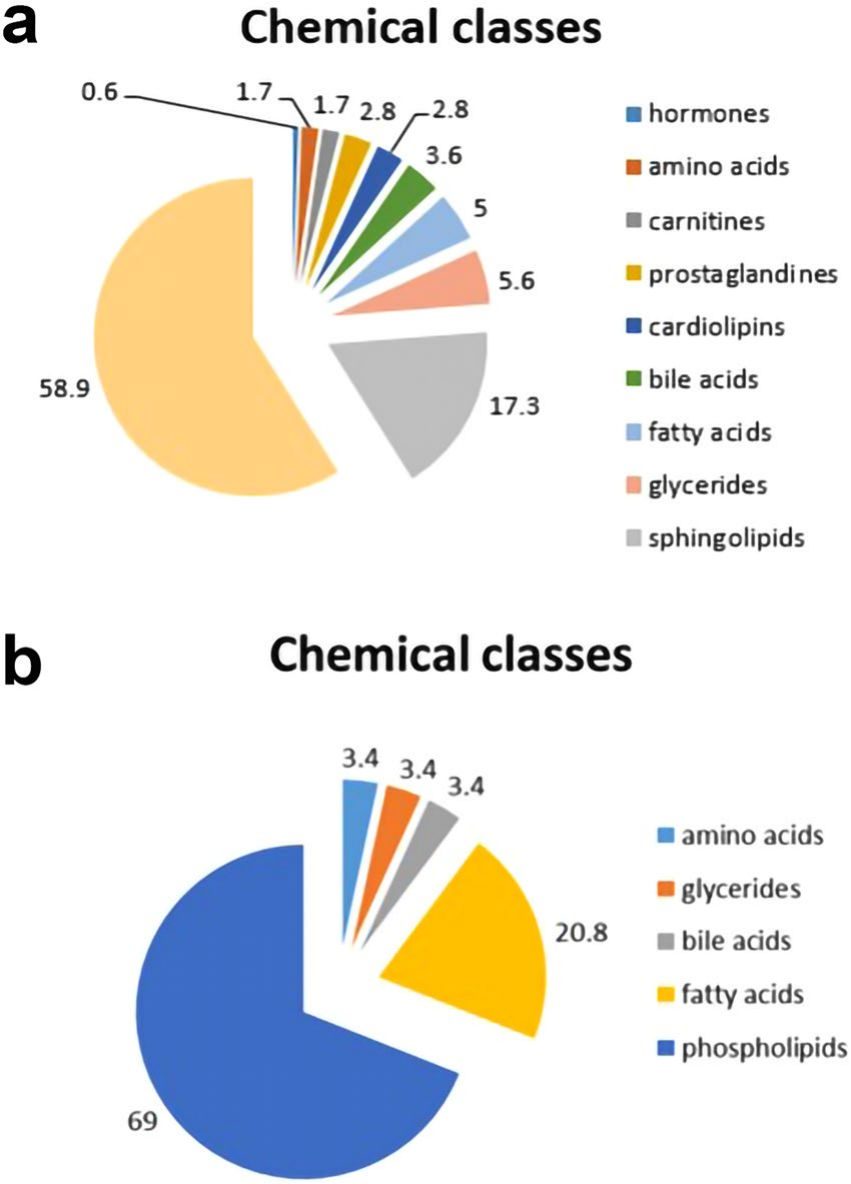
Chemical classes of putatively identified metabolites which were present only in plasma samples prepared by the procedure including incubation with proteinase K. Analytical measurements were performed with the use of the LC-ESI-TOF-MS technique in positive (**a**) and negative (**b**) ionization modes.

**Figure 4:**
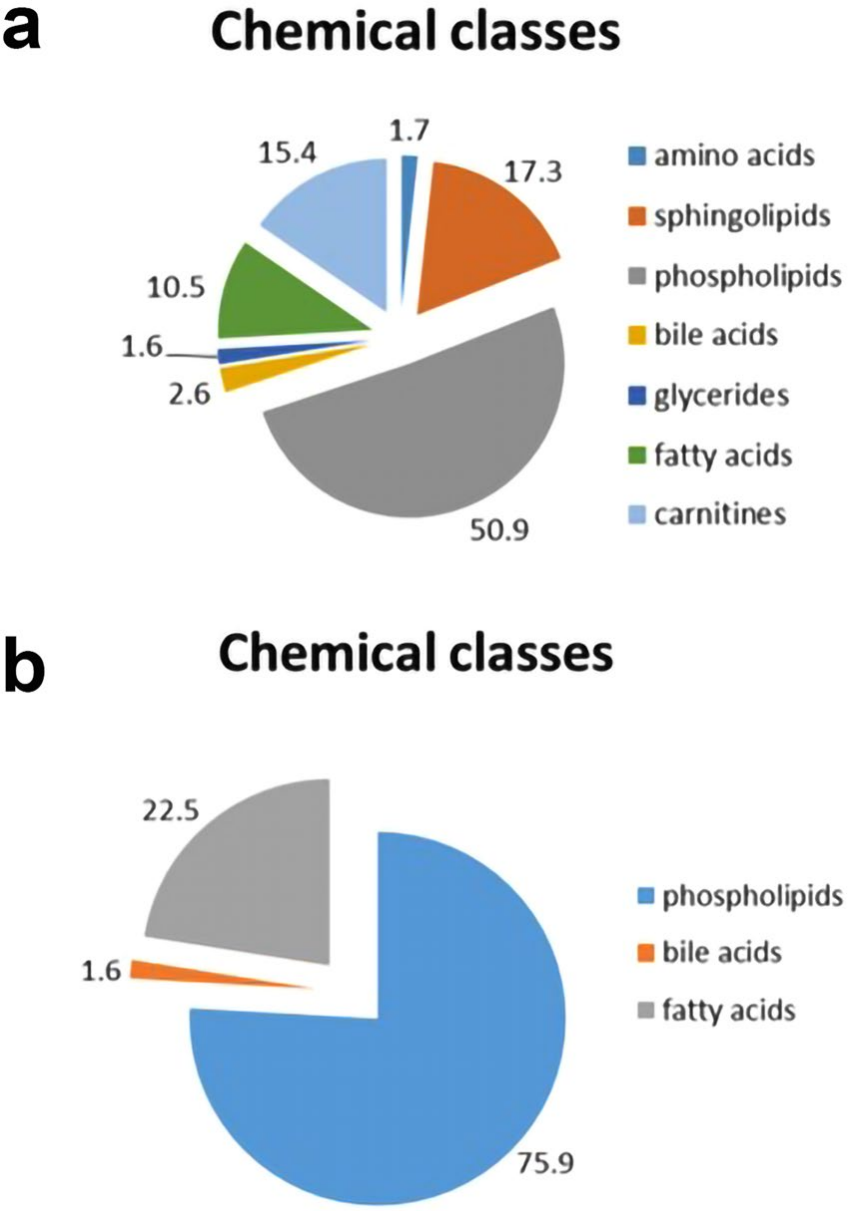
The chemical categorization of putatively identified metabolites representing higher abundance for the procedure with proteinase K. Plasma metabolic fingerprints were determined with the use of the LC-ESI-TOF-MS technique in positive (**a**) and negative (**b**) ionization modes.

The identified metabolites present only in plasma samples prepared by the procedure including incubation with PK were classified in terms of their chemical groups (Fig. 3). Additionally, the intensities of common features detected in plasma samples prepared with the use of both compared procedures were also verified and evaluated. The identified metabolites representing higher abundances for the procedure with proteinase K were categorized based on their chemical classes (Fig. 4).

### Application of the newly developed procedure for plasma samples derived from urogenital tract cancer patients

**Figure 5:**
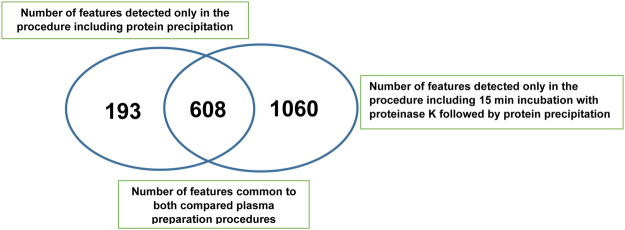
Venn diagram showing comparison of total number of features detected in plasma samples of urogenital tract cancer patients prepared using both tested procedures. Plasma metabolic fingerprints were obtained with the use of the LC-ESI-TOF-MS technique in both positive and negative ionization modes.

**Figure 6:**
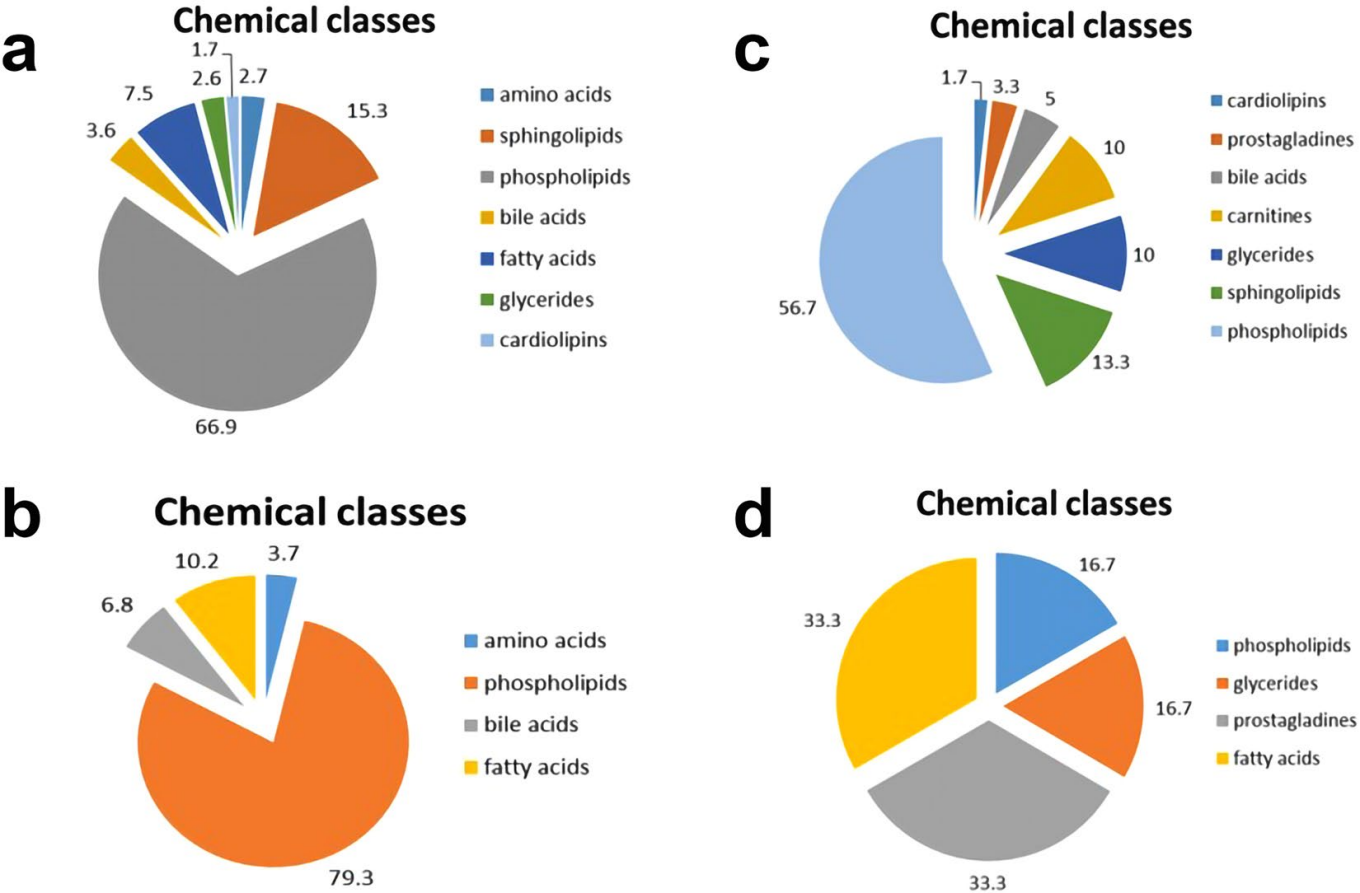
Chemical characterization of putatively identified metabolites which were present only in plasma samples prepared by procedure including incubation with proteinase K. Analytical measurements were performed with the use of the LC-ESI-TOF-MS technique in positive (**a**) and negative (**b**) ionization modes. The chemical classes of putatively identified metabolites representing at least twofold higher abundance for the procedure with proteinase K. Plasma metabolic fingerprints were determined with the use of the LC-ESI-TOF-MS technique in positive (**c**) and negative (**d**) ionization modes.

Based on previously described results, the new and optimized procedure including 15 min plasma incubation with proteinase K was applied to plasma samples (n = 10) derived from urogenital tract cancer patients and compared with the commonly used protein precipitation approach. The results of the comparison of plasma metabolic fingerprints obtained after application of both compared sample preparation procedures are presented in Figs 5–6 and Table 2.

**Table 2:** Comparison of results obtained for plasma metabolic fingerprints determined after preparation using both compared procedures.

	Protein precipitation with methanol:ethanol (1:1)	15 min incubation with proteinase K followed by protein precipitation with methanol:ethanol (1:1)
Total number of features present in all plasma samples (n = 10)	801	1668
Number of common features for both procedures with CV of their intensities < 20%	608	
Number of common features (CV < 20%) with at least twofold higher intensity in the case of proteinase K procedure	557	
Number of putatively identified endogenous metabolites	353	882

Analytical measurements were performed with the LC-ESI-TOF-MS technique in both positive and negative ionization modes.

## Discussion

### Recent analytical attempts to improve plasma metabolome coverage

Metabolome coverage in untargeted metabolomics still constitutes a crucial challenge. Metabolite extraction and protein precipitation with organic solvents is the most frequently applied procedure^7^. There are some previously reported studies comparing various organic solvents used for plasma sample preparation for metabolome coverage and method reproducibility^7,8,14–16^.

The most frequently applied organic solvents include acetonitrile, acetone, methanol, ethanol and their mixtures at various ratios^14,15^. Want *et al*. evaluated and tested 14 different extraction methods and observed that acid or heat treatments extracted significantly fewer compounds, whereas organic solvents, such as 30:70 acetone:methanol, 100% methanol and 50:50 acetone:methanol, provided the highest number of reproducible analytical signals, the broadest serum metabolome coverage as well as the highest efficiency of protein removal^15^. Additionally, plasma protein precipitation using methanol/ethanol (3609 features) ensured the best results for metabolite coverage and method precision as compared to *in vivo* SPME (1868 features) and ultrafiltration (2262 features) in LC-MS based untargeted metabolomics^17^. Dmitri *et al*. evaluated seven solvent-based and solid-phase extraction methods with the use of standard analytes spiked into both buffer and human plasma^14^. The parameters, such as recovery, coverage, repeatability, matrix effects, selectivity as well as the orthogonality of all methods tested, were evaluated and compared. The results obtained in the study showed a high degree of overlap, which resulted in metabolite coverage increases of 34–80% depending on the LC-MS method used as compared to the single extraction procedure (methanol/ethanol precipitation). However, the increase in MS analysis time and sample consumption should be also underlined. Moreover, ion exchange solid-phase extraction and liquid-liquid extraction with methyl-tertbutyl ether were observed to be the most orthogonal methods to methanol-based precipitation^14^.

Among recently published studies, some different approaches have aimed at improvements in plasma metabolome coverage in untargeted metabolomics^7,18–22^. Godzień *et al*. developed and applied a one-step extraction method using methyl-tert-butyl-ether consisting of a lipophilic and hydrophilic layer within a single vial insert, in-vial dual extraction (IVDE)^18^. In the case of the lipophilic phase, a 60 min lipid profiling method with LC-qTOF was developed and this enabled identification of fatty acids, glycerolipids, and glycerophospholipids as well as sterols. The aqueous phase of the obtained extract was analyzed with a previously reported method for plasma metabolic fingerprinting^23^. The proposed approach provides detection of over 4,500 metabolic features from a single 20 µl of plasma sample.

Yang *et al*. proposed and optimized a combined LL and SPE approach for LC-MS-based plasma profiling^19^. The new procedure was compared to PP using methanol and spiked internal standards as controls. As a result, five separate fractions enriched for aqueous species, phospholipids, fatty acids, neutral lipids, and hydrophobic lipids were obtained^19^. The combined approach demonstrated better reproducibility with CV’s below 15% for the combined procedure as compared to 30% observed for PP. Additionally, the proposed fractionation approach resulted in greater overall plasma metabolome coverage.

Skova *et al*. compared two different sample preparation techniques prior to LC-qTOF/MS-based plasma metabolic fingerprinting^20^. The first was PP and the second constituted PP followed by SPE resulted in three sub-fractions: phospholipid, lipid and polar. The proposed procedure was able to detect 4234 molecular features measured by the LC-qTOF/MS technique as compared to 1792 signals detected for the PP approach^20^. The proposed and optimized sample preparation procedure was applied for plasma samples collected from rats administered an environmental pollutant, namely perfluorononanoic acid.

Patterson *et al*. tested Folch, Bligh-Dyer, and Matyash lipid extractions^22^. Additionally, these methods were compared with the phospholipid removal plate procedure. Such a plate was used for lipid extraction and this has been proved to be a robust approach for targeted lipid analysis^22^. Folch and Matyash ensured reproducible recovery for a wide range of lipid classes; however, the Matyash aqueous layer was observed to be comparable with common methanol preparations for extraction and determination of polar metabolites. Therefore, the Matyash procedure was suggested as the best choice for biphasic extraction for untargeted plasma metabolomics and lipidomics^22^.

All the above-mentioned studies constitute recent attempts which have been undertaken to enhance plasma metabolomic profiles. Although improvements in metabolite coverage and good method reproducibility were observed, the proposed procedures are time-consuming and expensive, and include a lot of additional and difficult steps which can generate unwanted sources of analytical variability and therefore may be not suitable for analysis of larger set of biological samples. In the case of the commonly applied protein precipitation approach, the possible issue of co-precipitation still remains unaddressed in plasma untargeted metabolomics^24^.

In the present study, a new procedure with the use of a single additional step including limited digestion with proteinase K was developed and evaluated. The best results for the number of detected and reproducible analytical signals as well as the number of putatively identified metabolites were observed for 15 min plasma incubation with PK (Table 1). However, it should also be underlined that unspecific enrichment of peptide fragments can be observed in the obtained plasma metabolic fingerprints (Table 1). The new optimized sample preparation approach was applied to plasma samples collected from urogenital tract cancer and compared with methanol/ethanol precipitation previously observed as the most efficient and reproducible procedure (Table 2). The number of all detected analytical signals using proteinase K procedure was twice as high as that with the typical deproteinization approach (Table 2). Around 53% of these analytical signals were potentially annotated as endogenous metabolites. The putatively identified metabolites which were present only in plasma samples prepared with the use of proteinase K procedure representing mainly phospholipid, sphingolipid, fatty acid, bile acid, amino acid and cardiolipin classes. Additionally, for around 92% of common analytical signals detected in plasma metabolic profiles for both compared sample preparation approaches, at least twofold higher intensities were observed (Table 2). Among these analytical signals, metabolites belonging mainly to phospholipids, sphingolipids, carnitines, bile and fatty acids were tentatively identified.

The results of these preliminary studies indicate the beneficial effect of the new plasma sample preparation procedure including incubation with PK for metabolome coverage as well as the intensities of the signals derived from putatively identified endogenous metabolites. The proposed methodology, as compared to previously reported ones, is simple, fast, reproducible and not expensive or time-consuming; therefore, it seems to be a useful approach for plasma untargeted metabolomics which often require quite a large set of samples to provide biologically reliable results.

### The individual metabolites within the context of the biological processes

To find how the results of the optimized procedure could improve the biological and biomedical application of untargeted metabolomics analysis, we integrated the uniquely detected small metabolites (Table S2, Supplementary information) with biochemical pathway networks. Our results suggest that the optimized procedure allows the detection of unique crucial small metabolites and could provide an additional dimension of regulatory information. The KEGG pathway analysis (combined in Table S2, Supplementary information) assigned the proteinase K-procedure’s unique metabolites into several categories including: amino acids biosynthesis and metabolism, purine and pyrimidine metabolism, sphingolipid metabolism as well as pentose phosphate pathway, biosynthesis of terpenoids and steroids, and biosynthesis of secondary messengers. Although it is highly plausible to argue that the majority of these uniquely detected hydrophobic features were released from plasma albumin upon PK digestion, we cannot exclude the possibility that the identification of some phospholipids was in part a result of lipoprotein degradation. Indeed, our PK enriched procedure resulted in unique detection of phospholipids, such as phosphatidic acid (PA), lysophosphatidic acid (LPA), phosphatidylinositol (PI), diglyceride/diacylglycerol (DG/DAG), phosphatidylinositol bisphosphate (PIP2), and phosphatidylinositol trisphosphate (PIP3). It is highly plausible to suggest that their identification is a direct result of lipoprotein and plasma phospholipid transfer protein (PLTP) digestion^25–28^. Since plasma phospholipids levels are widely associated with cardiovascular diseases, our modified procedure could provide a novel initial metabolomic insight into these pathologies.

Furthermore, although we have observed enrichment in hydrophobic amino acids that resulted in assignment of the metabolites into protein degradation and absorption pathways, this enrichment could be partially related to PK activity.

Moreover, the procedure resulted in the detection of a large fraction of small metabolites that are usually assigned to plant (e.g., hexadecanedioate) and microbial metabolisms (e.g., ectoine). However, we cannot exclude the possibility that the presence of some of these features which are unusual for human metabolism could be a direct result of hemolysis.

## Conclusions

The majority of blood plasma proteins make contact with various small metabolites, and although these encounters may not always have functional consequences, it is obvious that for hydrophobic metabolites these interactions constitute a crucial transport and tissue delivery mechanism. Although achieving an *in vivo* global insight into these protein encounters fraction could provide important information and novel biomarkers for human pathologies, current metabolomic procedures focus mainly on circulating protein-unassociated small metabolites. As demonstrated in this study, a simple modification of the sample preparation procedure with a step allowing release of plasma protein- associated metabolites resulted in a significant enrichment of detected signals with individual features. The functional assignment of these metabolites covered a wide range of both anabolic and catabolic cellular processes (including hormones, aromatic amino acids and lipids). Although the performance and reproducibility of the new sample preparation approach should be tested and evaluated in a larger set of plasma samples, the modified protocol is simple, fast and easy to apply, and allows clear detection of hydrophobic metabolites including fatty acids and phospholipids. To the best of the authors’ knowledge, this is the first study to apply new sample preparation including addition of PK, which is the focus of the issue of metabolite co-precipitation, for LC-MS-based untargeted metabolomics. Taken together, the proposed methodology may provide an enrichment of plasma metabolome coverage mainly in terms of certain metabolite groups, such as phospholipids, sphingolipids, fatty and bile acids, acyl-carnitines and amino acids, and this would seem to be crucial in expanding current knowledge about the pathological processes of various diseases, for instance cancer and cardiovascular disorders.

## Electronic supplementary material

Supplementary information

## References

[1] Johnson CH, Ivanisevic J, Siuzdak G (2016). Metabolomics: beyond biomarkers and towards mechanisms. Nat Rev Mol Cell Biol..

[2] Zhou B, Xiao JF, Tuli L, Ressom WS (2012). LC-MS-based metabolomics. Mol Biosyst..

[3] Zhang A, Sun H, Wang P, Han Y, Wang X (2012). Modern analytical techniques in metabolomics analysis. Analyst..

[4] Schrimpe-Rutledge AC, Codreanu SG, Sherrod SD, McLean JA (2016). Untargeted Metabolomics Strategies-Challenges and Emerging Directions. J Am Soc Mass Spectrom..

[5] Rico E, González O, Blanco ME, Alonso RM (2014). Evaluation of human plasma sample preparation protocols for untargeted metabolic profiles analysed by UHPLC-ESI-TOF-MS. Anal Bioanal Chem.

[6] Vuckovic D (2012). Current trends and challenges in sample preparation for global metabolomics using liquid chromatography–mass spectrometry. Anal Bioanal Chem.

[7] Chen Z, Xu J, Zhang R, Abliz Z (2016). Methods used to increase the comprehensive coverage of urinary and plasma metabolomes by MS. Bioanalysis.

[8] Gika H, Theodoridis G (2011). Sample preparation prior to the LC–MS-based metabolomics/metabonomics of blood-derived samples. Bioanalysis.

[9] Li X, Gianoulis TA, Yip KY, Gerstein M, Snyder M (2010). Extensive *In vivo* Metabolite-Protein Interactions Revealed by Large-Scale Systematic Analyses. Cell..

[10] Schaller, J., Gerber, S., Kämpfer, U., Lejon, S. & Trachsel, C. *Human Blood Plasma Proteins: Structure and Function*. (John Wiley & Sons, Ltd, 2008).

[11] Ebeling W (1974). Proteinase K from Tritirachium album Limber. Eur J Biochem..

[12] Burrell, M. M. *Enzymes of Molecular Biology*. 307 (Humana Press, Totowa, New Jersey, 1993).

[13] Zamyslowska A (2017). Combination of LC−MS- and GC−MS-based metabolomics to study the effect of ozonated autohemotherapy on human blood. J Proteome Res..

[14] Sitnikov DG, Monnin CS, Vuckovic D (2016). Systematic Assessment of Seven Solvent and Solid-Phase Extraction Methods for Metabolomics Analysis of Human Plasma by LC-MS. Sci Rep..

[15] Bruce SJ (2009). Investigation of human blood plasma sample preparation for performing metabolomics using ultrahigh performance liquid chromatography/mass spectrometry. Anal Chem..

[16] Want EJ (2006). Solvent-dependent metabolite distribution, clustering, and protein extraction for serum profiling with mass spectrometry. Anal Chem..

[17] Vuckovic D (2011). *In vivo* solid phase microextraction: capturing the elusive portion of metabolome. Angew Chem Int Ed Engl..

[18] Godzien J (2013). In-vial dual extraction liquid chromatography coupled to mass spectrometry applied to streptozotocin-treated diabetic rats. Tips and pitfalls of the method. J Chromatogr A..

[19] Yang Y (2013). New sample preparation approach for mass spectrometry-based profiling of plasma results in improved coverage of metabolome. J Chromatogr A..

[20] Skova K, Hadrupb N, Smedsgaard J, Frandsen H (2015). LC–MS analysis of the plasma metabolome. A novel sample preparation strategy. J Chromatogr B..

[21] Kirkwood, J. S., Maier, C. & Stevens, J. F. Simultaneous, untargeted metabolic profiling of polar and nonpolar metabolites by LC-Q-TOF mass spectrometry. *Curr Protoc Toxicol*. **4**, 10.1002/0471140856.tx0439s56 (2013). 10.1002/0471140856.tx0439s56 PMC3707319 23670865

[22] Patterson RE, Ducrocq AJ, McDougall DJ, Garrett TJ, Yost RA (2015). Comparison of blood plasma sample preparation methods for combined LC-MS lipidomics and metabolomics. J Chromatogr B Analyt Technol Biomed Life Sci..

[23] Ciborowski M (2010). Metabolomic approach with LC-MS reveals significant effect of pressure on diver’s plasma. J Proteome Res..

[24] Vuckovic, D. Sample Preparation in Global Metabolomics of Biological Fluids and Tissues. *Proteomic and Metabolomic Approaches to Biomarker Discovery*. 51–76 (Academic Press, Elsevier, 2013).

[25] Schlitt A (2003). High plasma phospholipid transfer protein levels as a risk factor for coronary artery disease. Arterioscler Thromb Vasc Biol..

[26] Wang, Q. *et al*. Plasma phospholipid trans-fatty acids levels, cardiovascular diseases, and total mortality: the cardiovascular health study. *J Am Heart Assoc*. **27**, 10.1161/JAHA.114.000914 (2014). 10.1161/JAHA.114.000914 PMC4310377 25164946

[27] Upadhyay, R.K. Emerging risk biomarkers in cardiovascular diseases and disorders. *J Lipids*. **2015**, 10.1155/2015/971453 (2015). 10.1155/2015/971453 PMC4407625 25949827

[28] Cheung MC (2006). Phospholipid transfer protein activity is associated with inflammatory markers in patients with cardiovascular disease. Biochim Biophys Acta..

