# Supplementary material for: New plasma preparation approach to enrich metabolome coverage in untargeted metabolomics: plasma protein bound hydrophobic metabolite release with proteinase K
Source: Sci Rep. 2018 Jun 22;8:9541. doi: 10.1038/s41598-018-27983-0 (PMC6015025; doi:10.1038/s41598-018-27983-0)
Supplement: Supplementary file 1 — Supplementary information [file 41598_2018_27983_MOESM1_ESM.doc]

**Supplementary information**

New plasma preparation approach to enrich metabolome coverage in untargeted metabolomics: plasma protein bound hydrophobic metabolite release with proteinase K.

Renata Wawrzyniak ^1^, Anna Kosnowska ^1^, Szymon Macioszek ^1^, Rafał Bartoszewski ^2^ and Michał Jan Markuszewski ^1^*

^1^Department of Biopharmaceutics and Pharmacodynamics, Medical University of Gdańsk, Poland

^2^Department of Biology and Pharmaceutical Botany, Medical University of Gdańsk, Poland

*Corresponding author: Michał Jan Markuszewski

Tel.: +48 58 349 14 94/58 349 14 93; fax: +48 58 349 19 62.

E-mail address: [markusz@gumed.edu.pl](mailto:markusz@gumed.edu.pl)

**Materials and methods**

Analytical measurements

Chromatographic measurements were performed at the temperature of 35°C using a Zorbax Extend-C18 column (2.1 × 100 mm, 3.5 μm, Agilent Technologies, USA). Prepared plasma samples (2 μl) were analyzed using scan mode in the m/z range 50 to 1100 (mass to charge ratio) in both positive and negative ionization modes. The mobile phase consisted of 0.1% formic acid (97% FA, Alfa Aesar, Germany) in deionized water (A) and 0.1% FA in methanol (B). A flow rate of 0.5 ml/min was applied. The following gradient program was performed: from 0 to 20 min, mobile phase B eluted from 5% to 100% and then was kept for 7 min at 100% of B. An equilibration time of 5 min was used. The scan rate was set to 1.51 spectra/second. To provide accurate mass measurements, four reference masses (m/z 121.0509 and 922.0098 in the positive mode as well as m/z 112.9856 and 1033.9881 in the negative ionization mode) were automatically delivered using dual ESI source during plasma sample analyses. Capillary voltage was set to 3250 V for both ionization modes and the nebulizer gas flow rate and pressure were 10 l/min and 45 psig, respectively. Fragmentor voltage was set to 150 V.

**Results**

Table S1. Comparison of various organic solvents used for plasma protein precipitation and metabolite extraction. Plasma metabolic fingerprints were measured with the use of LC-ESI-TOF-MS techniques in both positive and negative ionization modes.

|  | **Acetonitrile** | **Methanol** | **Methanol:Ethanol**  **(1:1, v/v)** |
| --- | --- | --- | --- |
| **Number of features present in all plasma samples (n=6)** | **918** | **1453** | **1473** |
| **Number of features with CVs of their intensities <20% (n=6)** | **341** | **828** | **873** |

CV: coefficient of variation

Figure S1. Panel a presents EICs of compounds detected only after proteinase K treatment. In panel b new sample preparation method is compared with the standard procedure. Higher peaks indicate sample enrichment after additional step of incubation with proteinase K.


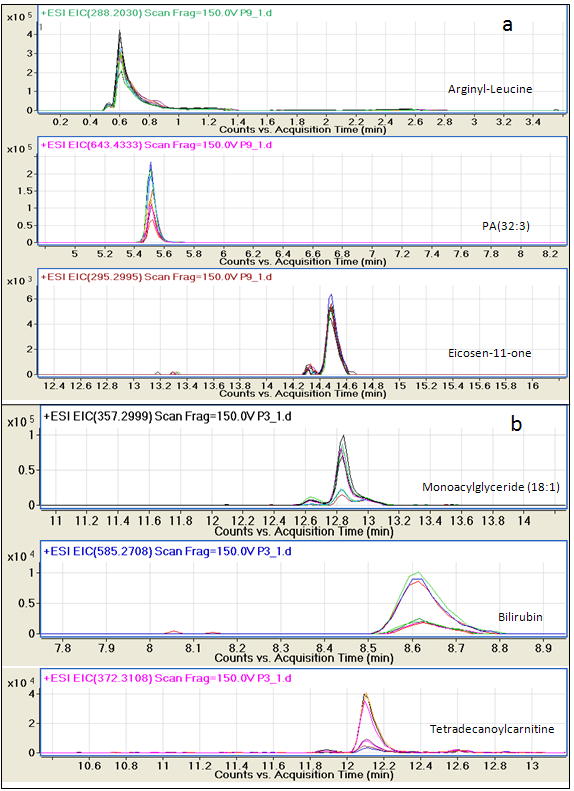


Table S2. Individual small metabolites integrated with biochemical pathway networks detected only in the case of proteinase K procedure.

| Metabolite | Metabolic pathway |
| --- | --- |
| Phospho-ribose diphosphate  Aminohexanoylaminohexanoate  Phenylacetamide  Hydroxybenzoyl-CoA  Diazacyclotetradecanedione  Ectoine  Hydroxyphenylpropanoic acid  Hydroxyectoine  Methylglutamylsemialdehyde-lysine  Glutamine | Microbial metabolism in diverse environments |
| Phospho- ribose diphosphate  Protoporphyrin  Hydroxybenzoyl-CoA | Biosynthesis of secondary metabolites |
| Phenylacetamide  Hydroxyphenylpropanoic acid | Phenylalanine metabolism |
| Aminohexanoylaminohexanoate  Diazacyclotetradecanedione | Caprolactam degradation |
| Ectoine  Hydroxyectoine | Glycine, serine and threonine metabolism |
| Phospho -ribose diphosphate | Phenylalanine, tyrosine and tryptophan biosynthesis |
| Hydroxybenzoyl-CoA  Hydroxyphenylpropanoic acid | Degradation of aromatic compounds |
| Acetyl-lysine  Lysopine | Lysine degradation |
| Hydroxybenzoyl-CoA | Aminobenzoate degradation  Ubiquinone and other terpenoid-quinone biosynthesis  Fluorobenzoate degradation  Benzoate degradation |
| Phospho-ribose diphosphate | Pentose phosphate pathway  Antifolate resistance  Biosynthesis of alkaloids derived from histidine and purine  Phenylalanine, tyrosine and tryptophan biosynthesis  Folate biosynthesis  Pyrimidine metabolism  Histidine metabolism  Purine metabolism  Carbon metabolism |
| Porphyrin | Neuroactive ligand-receptor interaction |
| Melibiitol | Galactose metabolism |
| Hexadecanedioate | Cutin, suberine and wax biosynthesis |
| Protoporphyrin | Porphyrin and chlorophyll metabolism |
| Phytosphingosine | Sphingolipid metabolism |
| Deoxymyxol-fucoside | Carotenoid biosynthesis |
| Dihydroxyindole | Tyrosine metabolism |
| Methylglutamyl-semialdehyde-lysine | Lysine biosynthesis |
| Phenylacetamide | Styrene degradation |
| Diacetamido-trideoxy-mannopyranose | Amino sugar and nucleotide sugar metabolism |
| Deoxymyxol-fucoside | Carotenoid biosynthesis |
| Methylerythritol phosphate | Terpenoid backbone biosynthesis  Biosynthesis of terpenoids and steroids  Biosynthesis of secondary metabolites |
| Tri(hydroxyferuloyl) spermidine | Phenylpropanoid biosynthesis |

| **Non-specific metabolite changes probably related to the activity of proteinase K** | |
| --- | --- |
| Metabolite | Metabolic pathway |
| Methionine  Phenylalanine  Leucine  Piperidine  Glutamine | Protein digestion and absorption |
| Methionine  Phenylalanine  Phospho-ribose diphosphate  Leucine  Glutamine | Biosynthesis of amino acids |
| Methionine  Phenylalanine  Leucine  Glutamine | 2-Oxocarboxylic acid metabolism  Mineral absorption  Glucosinolate biosynthesis  Aminoacyl-tRNA biosynthesis  Central carbon metabolism in cancer |

Table S3. Putatively identified features. Formula match score was calculated by Agilent Mass Hunter Qualitative Analysis on the basis of measured mass, abundance pattern of the isotope cluster and isotope spacing. Ppm error – difference between experimental and theoretical mass. Fragments were observed in mass spectra in corresponding retention time. Identification level according to the Metabolomics Standards Initiative.

| **Compound** | **Ionisation mode** | **RT [min]** | **Experimental mass** | **Ppm error** | **Formula match score** | **Observed fragments [m/z]** | **Identification level** |
| --- | --- | --- | --- | --- | --- | --- | --- |
| Alanyl-Isoleucine or  Alanyl-Leucine | + | 1.71 | 202.1321 | 2 | 98 % | 157.1335 | 2 |
| Tryptophan | + | 3.53 | 204.0901 | 1 | 98 % | 188.0696 | 2 |
| C16-Sphinganine | + | 6.39 | 273.2667 | 0 | 82 % | 256.2630 | 2 |
| Dodecanoylcarnitine | + | 6.80 | 343.2718 | 1 | 89 % | - | 3 |
| C18-Sphinganine | + | 7.6 | 301.2977 | 0 | 85 % | 282.2797 | 2 |
| PE(20:4) | + | 11.26 | 501.2803 | 1 | 88 % | - | 3 |
| 2-methoxy-6-hexadecenoic acid | + | 11.28 | 284.2354 | 1 | 93 % | - | 3 |
| PG(20:5) | + | 11.95 | 530.2671 | 5 | 92 % | 285.221 | 2 |
| Octadecenal | + | 14.07 | 266.2611 | 0 | 98 % | 249.2580 | 2 |
| PC(40:5) | + | 14.24 | 835.6078 | 2 | 87 % | - | 3 |
| Alanyl-Phenylalanine | - | 2.92 | 236.1168 | 3 | 92 % | - | 3 |
| PG(38:9) | - | 4.31 | 788.4641 | 2 | 90 % | - | 3 |
| Arachidonic acid | - | 12.75 | 304.2404 | 1 | 93 % | - | 3 |
| Palmitic acid | - | 13.32 | 256.2406 | 1 | 92 % | - | 3 |
| PS(35:0) | - | 16.05 | 777.5504 | 2 | 89 % | - | 3 |
